# Roles of B739_1343 in iron acquisition and pathogenesis in *Riemerella anatipestifer* CH-1 and evaluation of the RA-CH-1*ΔB739_1343* mutant as an attenuated vaccine

**DOI:** 10.1371/journal.pone.0197310

**Published:** 2018-05-30

**Authors:** MaFeng Liu, Mi Huang, Yun Shui, Francis Biville, DeKang Zhu, MingShu Wang, RenYong Jia, Shun Chen, KunFeng Sun, XinXin Zhao, Qiao Yang, Ying Wu, XiaoYue Chen, AnChun Cheng

**Affiliations:** 1 Institute of Preventive Veterinary Medicine, College of Veterinary Medicine of Sichuan Agricultural University, Chengdu, Sichuan, P.R. China; 2 Research Center of Avian Disease, College of Veterinary Medicine of Sichuan Agricultural University, Chengdu, Sichuan, P.R. China; 3 Key Laboratory of Animal Disease and Human Health of Sichuan Province, Chengdu, Sichuan, P. R. China; 4 Unité des Infections Bactériennes Invasives, Département Infection et Epidémiologie, Institut Pasteur, Paris, France; The University of Melbourne, AUSTRALIA

## Abstract

Iron is one of the most important elements for bacterial survival and pathogenicity. The iron uptake mechanism of *Riemerella anatipestifer* (*R*. *anatipestifer*, RA), a major pathogen that causes septicemia and polyserositis in ducks, is largely unknown. Here, the functions of the putative TonB-dependent iron transporter of RA-CH-1, B739_1343, in iron utilization and pathogenicity were investigated. Under iron-starved conditions, the mutant strain RA-CH-1*ΔB739_1343* exhibited more seriously impaired growth than the wild-type strain RA-CH-1, and the expression of *B739_1343* in the mutant strain restored growth. qRT-PCR results showed that the transcription of *B739_1343* was not regulated by iron conditions. In an animal model, the median lethal dose (LD_50_) of the mutant strain RA-CH-1*ΔB739_1343* increased more than 10^4^-fold (1.6×10^12^ CFU) compared to that of the wild-type strain RA-CH-1 (1.43×10^8^ CFU). In a duck co-infection model, the mutant strain RA-CH-1*ΔB739_1343* was outcompeted by the wild-type RA-CH-1 in the blood, liver and brain of infected ducks, indicating that B739_1343 is a virulence factor of RA-CH-1. Finally, immunization with live bacteria of the mutant strain RA-CH-1*ΔB739_1343* protected 83.33% of ducks against a high-dose (100-fold LD_50_) challenge with the wild-type strain RA-CH-1, suggesting that the mutant strain RA-CH-1*ΔB739_1343* could be further developed as a potential live attenuated vaccine candidate for the duck industry.

## Introduction

Iron is an essential element for most bacteria [1, 2]. The ability of pathogenic bacteria to obtain iron from their host is a key determinant of virulence [3]. Conversely, to protect against invading pathogens that steal iron, hosts sequester iron using host iron-binding proteins, such as transferrin, lactoferrin and ferritin, and hemin-containing protein, such as hemoglobin, hemopexin, myoglobin and leghemoglobin [4]. To overcome host iron-withholding defenses, most bacterial pathogens have evolved highly sophisticated systems to acquire iron for successful infection [5, 6].

*Riemerella anatipestifer* (*R*. *anatipestifer*, RA) is the etiological agent of acute septicemia and infectious polyserositis in ducks, chickens, geese, and other avian species [7]. *R*. *anatipestifer* infection can give rise to high contagiosity and mortality among farm ducks, resulting in major economic losses in the poultry industry [8]. According to research reports, at least 21 serotypes of *R*. *anatipestifer* without cross-protection have been identified around the world [9–12]. Among clinically isolated strains, *R*. *anatipestifer* serotypes 1, 2 and 10 are the most prevalent, with a high level of virulence in China [13]. However, little is known about the pathogenicity or virulence of this pathogen based on nutritional metabolism associated with iron acquisition. The TonB-dependent receptor TbdR1 (Riean_1607) of *R*. *anatipestifer* strain CH3 has been reported to be involved in iron acquisition and virulence [14]. The ferric iron utilization gene B739_1208 of *R*. *anatipestifer* CH-1 was also recently shown to be involved in virulence [15]. However, the *R*. *anatipestifer* genome encodes at least 31 TonB-dependent receptors (TbdRs) [14], and the physiological roles of these putative TbdRs are mostly unknown.

Here, B739_1343 is demonstrated to be required for *R*. *anatipestifer* CH-1 growth under iron-limited conditions. Investigation of the virulence of *R*. *anatipestifer* RA-CH-1*ΔB739_1343* showed that this strain is attenuated in ducks and outcompeted by the wild-type strain RA-CH-1. Furthermore, an examination of protective efficacy revealed that the mutant can be used as a live attenuated vaccine candidate for protecting ducks from *R*. *anatipestifer* infection.

## Results

### *In silico* analyses of the *R*. *anatipestifer B739_1343* gene

It was shown that *R*. *anatipestifer* GSM15868 encodes at least 31 putative TbdRs [14]. The homologues of them are present in the genome of *R*. *anatipestifer* CH-1, one of which is *B739_1343*. B739_1343 of *R*. *anatipestifer* CH-1 has low identity with TbdR1 of *R*. *anatipestifer* CH-3 [14] (7.29% identity) and B739_1208 of *R*. *anatipestifer* CH-1 [15] (3.4% identity). Similar to other characterized outer membrane iron transporters, the N-terminal region of B739_1343 contains a putative TonB interaction site (ETVVV, residues 95–99), and the conserved structure of the protein comprises a beta-barrel and a plug domain occupying the pore inside the barrel [16, 17]. According to the NCBI, the *B739_1343* protein is annotated as an outer membrane receptor involved in inorganic ion utilization. Additionally, the results of BLAST analyses revealed that the B739_1343 protein sequence shares over 98% identity in all sequenced *R*. *anatipestifer* strains, indicating that this sequence is highly conserved among different *R*. *anatipestifer* isolates.

### The mutant strain RA-CH-1*ΔB739_1343* exhibits reduced iron utilization in iron-starved conditions

**Fig 1:**
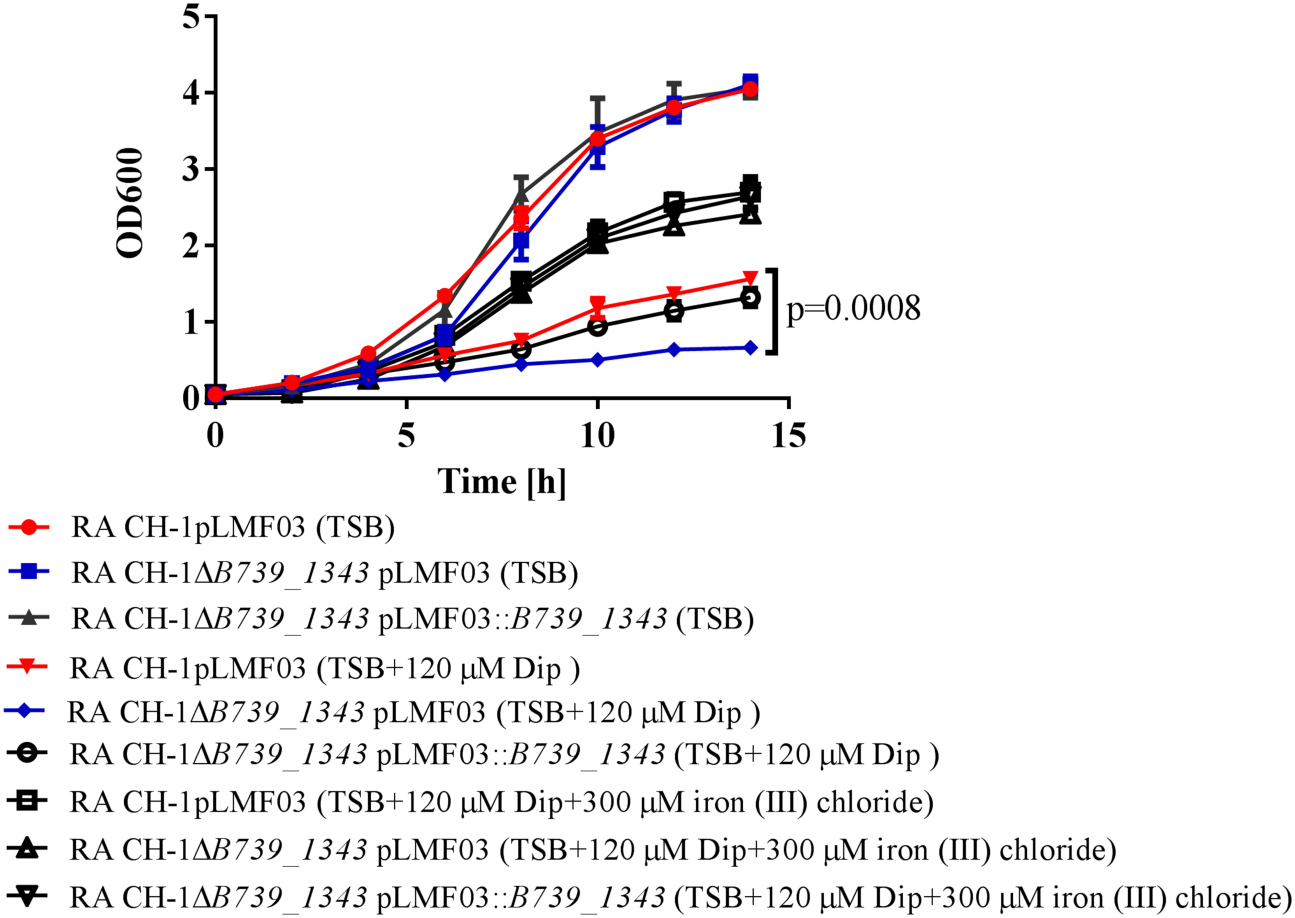
Growth curves for RA-CH-1pLMF03, RA-CH-1*ΔB739_1343*pLMF03, and RA-CH-1*ΔB739_1343*pLMF03::*B739*_*1343* in TSB, TSB supplemented with 120 μM Dip and TSB supplemented with 120 μM Dip and 300 μM iron (III) chloride. Cells were grown in 20 mL of TSB medium or TSB medium supplemented with Dip or TSB medium supplemented with Dip and iron (III) chloride at 37°C, starting at OD600 = 0.1. OD600 values were measured every 2 h for 14 h. Data were analyzed using two-way ANOVA. The error bars represent the standard deviations of three independent experiments and two replicate samples for each experiment (n = 3).

To identify the function of B739_1343, the mutant strain RA-CH-1*ΔB739_1343* was constructed and verified as described in the “Materials and Methods” section. After the *B739_1343* gene was knocked out, its ability to affect the growth of *R*. *anatipestifer* CH-1 in tryptone soy broth (TSB) liquid medium was evaluated. As shown in Fig 1, disruption of *B739_1343* did not damage the growth of *R*. *anatipestifer* CH-1 in TSB liquid medium. Moreover, the addition of the iron chelator 2,2'-dipyridyl at a final concentration of less than 80 μM impaired the optimal growth of both RA-CH-1*ΔB739_1343* and the wild-type strain at the same level (data not shown). However, the addition of the iron chelator 2,2'-dipyridyl at a final concentration of 120 μM impaired the maximal growth rate, e.g., the slope of the log-transformed growth curve during the exponential phase, of RA-CH-1*ΔB739_1343* more significantly than that of wild type. Complementation of RA-CH-1*ΔB739_1343* with a plasmid expressing *B739_1343* restored growth to wild-type levels. In addition, although *R*. *anatipestifer* CH-1 and RA-CH-1*ΔB739_1343* did not grow well in TSB containing 120 μM Dip, the addition of 300 μM iron (III) chloride to cultures restored the growth of all the strains (Fig 1). The two strains expressed equivalent levels of the downstream gene (*B739_1342*) of *B739_1343* (S1 Fig), confirming that the growth defect of RA-CH-1*ΔB739_1343* was due to a specific *B739_1343* mutation rather than a polar effect on gene expression levels. Overall, these data suggest that B739_1343 plays a pivotal role in *R*. *anatipestifer* CH-1 iron acquisition under iron starvation.

**Fig 2:**
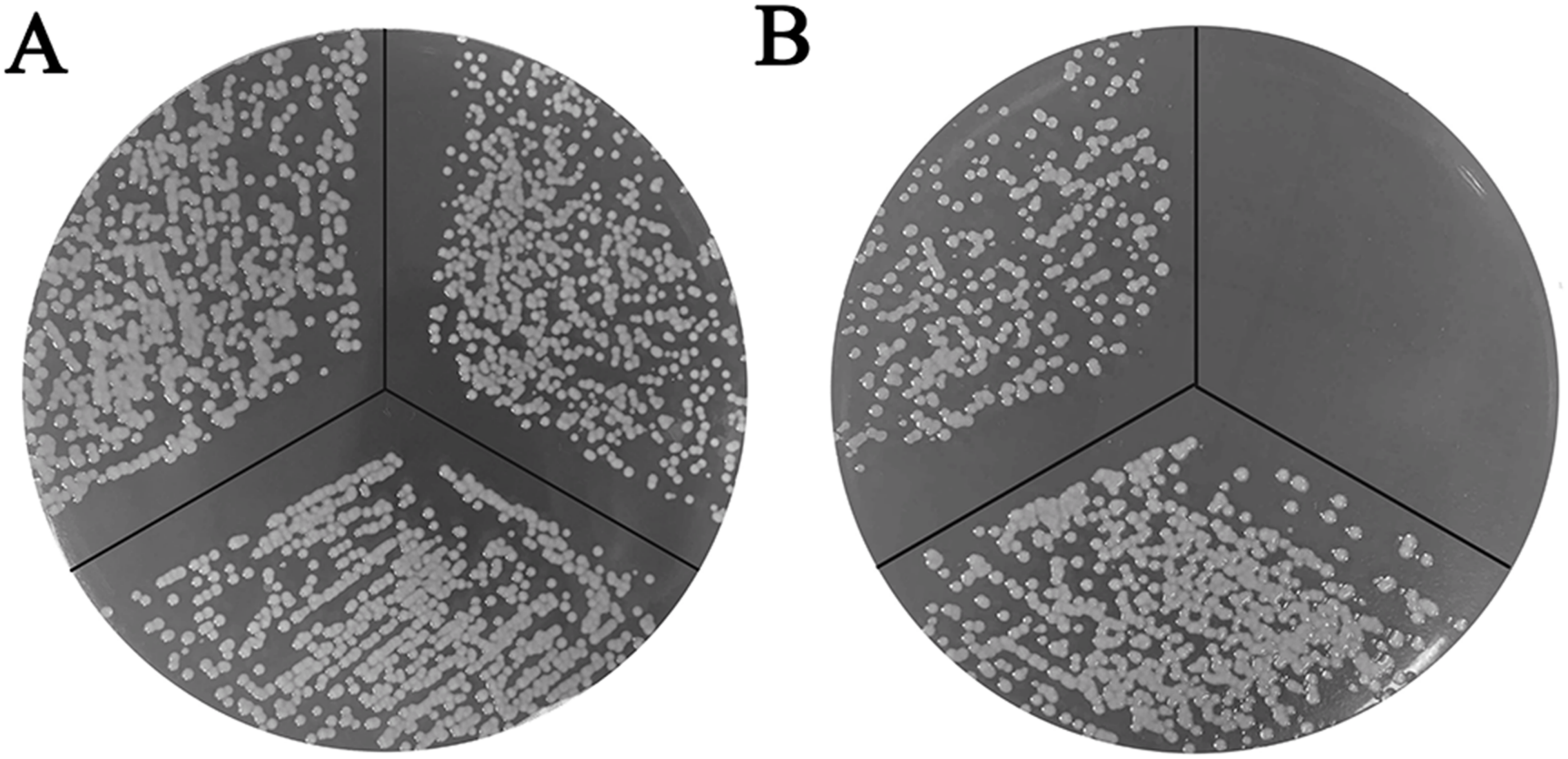
Growth of RA-CH-1pLMF03, RA-CH-1*ΔB739_1343*pLMF03, and RA-CH-1*ΔB739_1343*pLMF03::*B739_1343* on TSA and TSA supplemented with 50 μM Dip. The *R*. *anatipestifer* strains (clockwise from top left) RA-CH-1pLMF03, RA-CH-1*ΔB739_1343*pLMF03, and RA-CH-1*ΔB739_1343*pLMF03::*B739-1343* were grown on TSA plates containing cefoxitin (1 μg/mL) and 0 μM Dip (A) or 50 μM Dip (B). Growth was assessed by the appearance of bacterial colonies on plates. Pictures were taken after 48 h of growth at 37°C. All the experiments were repeated three times. Representative plates are presented.

To obtain further support for this hypothesis, the bacteria were also tested for growth on TSA plates with or without Dip. As shown in Fig 2A, deletion of *B739_1343* had no effect on the growth of the bacteria on TSA plates. However, *R*. *anatipestifer* CH-1*ΔB739_1343*pLMF03 was not able to grow on TSA plates containing 50 μM Dip, and the RA-CH-1*ΔB739_1343*pLMF03::*B739_1343* complementation strain restored growth (Fig 2B). These results obtained on a solid medium agree with those obtained in a liquid medium, indicating that the *B739_1343* gene of *R*. *anatipestifer* CH-1 is involved in ferric iron utilization.

### The transcription of *B739_1343* is not regulated by iron

In other bacteria, such as *Vibrio vulnificus* and *Corynebacterium diphtheriae*, iron uptake-related genes are negatively regulated by iron [18–21]. Thus, we sought to determine whether *B739_1343* is also regulated according to this model. The transcription levels of *B739_1343* in TSB (iron-rich medium) and TSB supplemented with 200 μM Dip (iron-depleted medium) were measured via qRT-PCR as described in the “Materials and Methods” section. The results showed no significant effect on the transcription of *B739_1343* under iron depletion (S2 Fig). However, the control gene, *B739_0103*, was upregulated ~30-fold under the same conditions (S2 Fig). Sequence analysis showed that the *B739_0103* promoter region possesses classic *R*. *anatipestifer* Fur boxes (ATTTATTTTTATTCTAAAT) [12], whereas the promoter of *B739_1343* does not. These results are consistent with the results of a previously reported RNA-Seq analysis of RA-CH-1 in TSB and TSB supplemented with Dip [22].

### The B739_1343 deletion attenuates the virulence of RA-CH-1 in ducklings

B739_1343 is involved in iron utilization by RA-CH-1; therefore, it was hypothesized that B739_1343 may also be involved in the virulence of the *R*. *anatipestifer* CH-1 strain. Thus, the LD_50_ was evaluated by infecting 3-day-old ducklings with RA-CH-1pLMF03, RA-CH-1*ΔB739_1343*pLMF03 and RA-CH-1*ΔB739_1343*pLMF03::*B739_1343*, as described in the “Materials and Methods” section. The mortality of the ducks was observed for 7 days post-challenge. The calculated LD_50_ value of the RA-CH-1*ΔB739_1343* mutant was 1.6×10^12^ CFU, whereas the LD_50_ value of RA-CH-1pLMF03 was 1.43×10^8^ CFU. The LD_50_ of the complementation strain was 8.66×10^9^ CFU, indicating that the *B739_1343* gene plays an important role in the virulence of *R*. *anatipestifer* CH-1.

### B739_1343 contributes to the colonization of *R*. *anatipestifer* CH-1 *in vivo*

**Fig 3:**
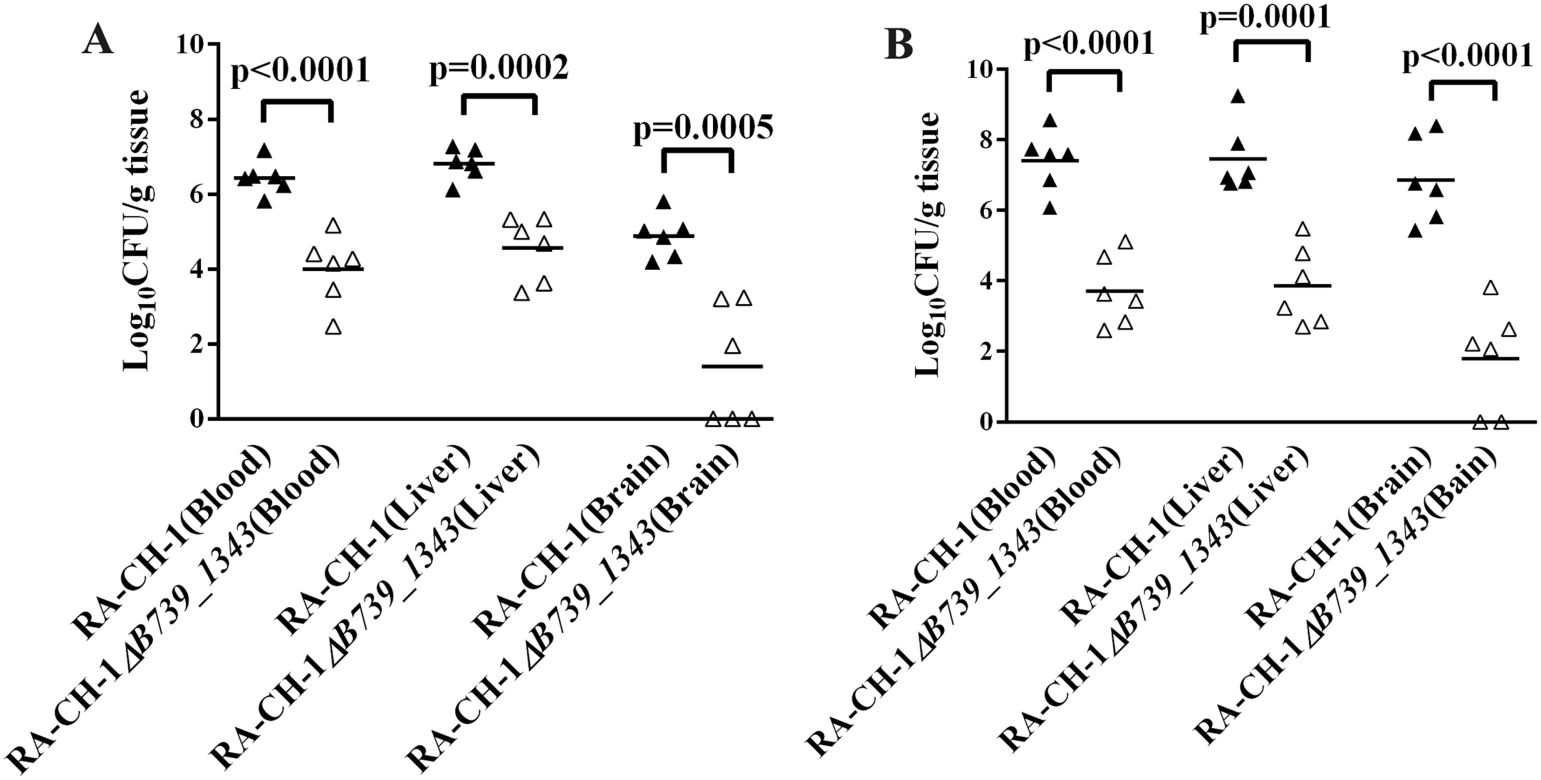
Competition assay of wild-type RA-CH-1 (filled symbols) and the mutant strain RA-CH-1*ΔB739_1343* (open symbols) *in vivo*. The wild-type strain RA-CH-1 (10^9^ CFU) and RA-CH-1 *ΔB739_1343* (10^9^ CFU) were mixed at a 1:1 ratio and injected into the leg muscles of 3-day-old ducklings. At 24 h (A) and 48 h (B) post-infection, bacteria were isolated from the livers, brains and blood according to the method described in the “Materials and Methods” section. The data points represent the CFU/g of individual animals in the indicated organs; the bars show the median values (n = 6).

To further investigate whether B739_1343 contributes to the colonization dynamics of *R*. *anatipestifer* during systemic infection, a competitive experiment was conducted in which each duck was subjected to intramuscular inoculation with a 1:1 ratio of the wild-type strain RA-CH-1 and the mutant strain RA-CH-1*ΔB739_1343*, as described in the “Materials and Methods”. At 24 h post-inoculation, the bacterial load of the mutant strain in the heart blood, livers and brains of the ducks was significantly reduced compared with that of the wild-type strain (Fig 3A). At 48 h post-inoculation, the difference in the bacterial load between the wild-type strain and the mutant strain was more apparent in the heart blood (5×10^3^-fold reduction, P<0.0001), liver (3.9×10^3^-fold reduction, P = 0.0001) and brain tissue (10^5^-fold reduction, P<0.0001) (Fig 3B). Thus, the RA-CH-1*ΔB739_1343* mutant was significantly outcompeted by the RA-CH-1 wild-type strain in these tissues, suggesting that *B739_1343* contributes to the ability of *R*. *anatipestifer* CH-1 to colonize the blood and liver and disseminate to the brain.

### Immunization and determination of serum antibody levels

Since the mutant strain RA-CH-1*ΔB739_1343* showed significantly attenuated pathogenicity, the potential use of RA-CH-1*ΔB739_1343* as a live attenuated vaccine against infection by the virulent RA-CH-1 wild-type strain was evaluated. First, injection of the mutant strain was evaluated to determine whether it affected the health of ducklings. Ducklings immunized with RA-CH-1*ΔB739_1343* showed no visible differences in habits or appetites compared with uninjected ducklings. The average body weights and body weight gains during the observation period of the ducklings immunized with RA-CH-1*ΔB739_1343* (group 2) did not differ significantly from those of the ducks in the group injected with PBS (group 1), the group immunized with the inactivated RA-CH-1 vaccine (group 3) or the group receiving no immunization (group 4) (Table 1). Thus, the mutant strain RA-CH-1*ΔB739_1343* is a suitable attenuated vaccine candidate.

**Table 1:** Duck body weight before challenge and body weight gain (mean±SD).

Group	Immunization	Body weight before vaccination (g)^a^	Body weight gain after vaccination (g)			
			D3	D6	D9	D12
1	PBS	86.25±7.02 (p = 0.9264)	66.1±5.38 (p = 0.1236)	141.1±11.48 (p = 0.5214)	245.4±19.97 (p = 0.2792)	331.9±27.01 (p = 0.3925)
2	RA-CH-1*ΔB739*_*1343*	85.65±7.6 (p = 0.9936)	66.85±5.94 (p = 0.1154)	143.8±12.77 (p = 0.3988)	229.5±20.37 (p = 0.8611)	337.1±29.93 (p = 0.4647)
3	Inactivated RA-CH-1 vaccine	85.5±7.54 (p = 0.9742)	61.1±5.39 (p = 0.5150)	141±12.44 (p = 0.5452)	229.9±20.28 (p = 0.8419)	326.1±28.77 (p = 0.3034)
4	-	85.7±6.67	58.2±4.53	134.8±10.48	226.6±17.62	353.2±27.47

^a^Number of ducks in a group (n = 20).

**Fig 4:**
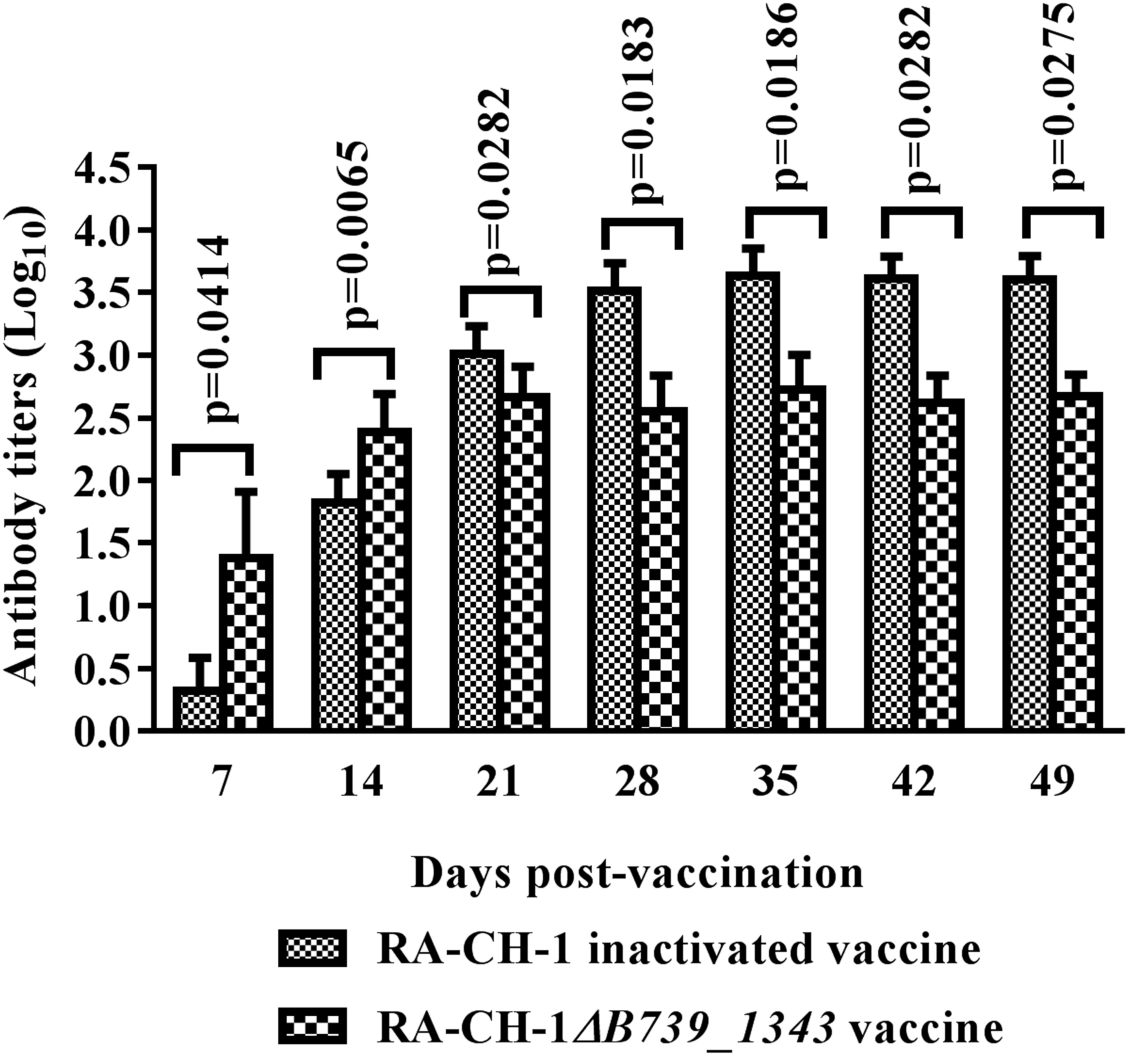
Serum antibody titers of ducks vaccinated with RA*-*CH-1*ΔB739_1343* and the inactivated RA-CH-1 vaccine (n = 5). Serum was collected from the ducks at 7-day intervals from day 7 to day 49 post-vaccination, and serum antibodies against RA*-*CH-1 were quantified using indirect ELISA. The antibody titers represent the highest dilutions that produced positive results. The data represent the average value from five serum samples for each group. The statistical significance of the data was ascertained with Student’s T test. This experiment was performed using three independent experiments and three replicate samples in each experiment (n = 3).

Next, the ducks inoculated with RA-CH-1*ΔB739_1343* were evaluated for a specific humoral immune response. To determine the serum antibody response after vaccination, serum samples were collected from vaccinated ducks at days 7, 14, 21, 28, 35, 42, and 49 after primary immunization, and serum antibodies against *R*. *anatipestifer* CH-1 were tested using indirect ELISA, as described previously [23]. As a control, negative sera samples were also collected from pre-immunized ducks. As shown in Fig 4, starting on day 7 and day 14 after immunization, the RA-CH-1*ΔB739_1343* vaccine elicited higher IgY titers than the inactivated RA-CH-1 vaccine (Fig 4). Furthermore, the serum antibody levels of both the RA-CH-1*ΔB739_1343* vaccine group and the inactivated RA-CH-1 group remained high for at least 49 days after immunization. However, the IgY antibody titer elicited by inactivated RA-CH-1 was higher than that elicited by RA-CH-1*ΔB739_1343* at day 21 (Fig 4). These results suggested that the RA-CH-1*ΔB739_1343* vaccine enhanced the specific humoral immune response in ducks.

### Evaluation of RA-CH-1*ΔB739_1343* as an attenuated vaccine

To examine whether the immunization of ducks with RA-CH-1*ΔB739_1343* can prevent infection by wild-type RA-CH-1, challenge experiments were performed as described in the “Materials and Methods” section. After challenge with the wild-type strain RA-CH-1, the survival rates of the ducks in groups 1, 2 and 3 were 25%, 85% and 100%, respectively (Table 2). The surviving ducks in groups 2 and 3 remained healthy, whereas three of the five surviving ducks in group 1 showed clinical signs that included loss of weight and appetite, opisthotonus and drowsiness. Thus, the morbidity of group 1 was 90% (Table 2). After challenge, the average body weights and body weight gains of groups 2, 3 and 4 did not differ significantly (data not shown). Given the above results, RA-CH-1*ΔB739_1343*-vaccinated ducks were 83.3% protected from challenge by the RA-CH-1 wild-type strain, indicating that the RA-CH-1*ΔB739_1343* attenuated mutant strain can be used as a live vaccine candidate.

**Table 2:** Animal challenge experiment.

Group	Immunization	Challenge strain^a^	No. of deaths (total)	No. showing morbidity (total)	Mortality (%)	Morbidity (%)	Protection^b^ (%)
1	PBS	RA-CH-1	15(20)	18(20)	75%	90%	-
2	RA-CH-1*ΔB739*_*1343*	RA-CH-1	3(20)	3(20)	15%	15%	83.33%
3	Inactivated RA-CH-1 vaccine	RA-CH-1	0(20)	0(20)	0	0	100%
4	-	-	0(20)	0(20)	0	0	-

^a^The challenge strain dose was 100 LD_50_.

^b^The protection rate was calculated as [1−(% Morbidity in vaccinate/% Morbidity in control)]×100

## Discussion

Iron acquisition is an important aspect of pathogenesis for many pathogens, and bacterial strategies for the acquisition of iron have been described for many decades [24]. It has been shown that the inactivation of genes involved in iron acquisition in *Shigella* [25], *Y*. *pestis* [26], and *P*. *luminescens* [27] attenuates virulence in animal infection models. However, relatively little is known about the iron uptake machinery and the role that iron plays in the physiology and virulence of *R*. *anatipestifer*. The major objective of this study was to explore the role of B739_1343 (annotated as a putative TonB-dependent iron transporter) in the ferric iron utilization and pathogenesis of *R*. *anatipestifer* CH-1.

Our previous research showed that knockout of the ferric iron utilization gene B739_1208 in RA-CH-1 impaired growth in both iron-rich and iron-limited media [15]. Similarly, knockout of the ferric iron utilization gene *tbdR1* (Riean_1607) in *R*. *anatipestifer* strain CH3 also impaired growth in both iron-rich and iron-limited media [14]. In contrast, B739_1343 deletion affected growth only in iron-limited conditions, suggesting that there are multiple putative TonB-dependent iron transporters involved in iron utilization in RA-CH-1. The components of the iron uptake system and the mechanism involved are under investigation. Nevertheless, through growth analysis, we demonstrated that iron is required for optimal RA-CH-1 growth and that B739_1343 is important for iron uptake in an iron-limited environment. These conclusions were strengthened by the results of the growth assay on iron-limited TSA plates. However, in contrast to classic iron utilization-related genes, the transcription of B739_1343 was not upregulated under iron-limited conditions. The “Fur box” of *R*. *anatipestifer* was recently identified [12] but was not found in the promoter region of B739_1343. In addition to B739_1208 [15], B739_1343 is the second iron utilization-related gene that is not regulated by iron.

For most bacterial pathogens, including *R*. *anatipestifer*, iron acquisition in an iron-limited host is critical to virulence during infection [15, 28]. In *R*. *anatipestifer* CH-3, mutation of the putative TonB-dependent iron transporter *tbdR1* attenuated virulence by approximately 45-fold [14], whereas mutation of the siderophore-interacting protein *sip* attenuated virulence by approximately 35-fold [28]. In *R*. *anatipestifer* CH-1, the putative TonB-dependent iron transporter *B739_1208* was attenuated approximately 15-fold [15]. In this study, the LD_50_ of the RA-CH-1*ΔB739_1343* mutant was found to be increased by more than 10^4^-fold in a duck infection model, and expression of the *B739_1343* gene provided a competitive advantage for the colonization of tissues in a duck co-infection model. Thus, B739_1343 is likely to play a more important role than other TbdRs in the virulence of *R*. *anatipestifer*, although without any growth effect in an iron-rich environment.

To evaluate whether the *B739_1343* mutant can serve as an attenuated vaccine candidate, different doses of the *B739_1343* mutant were inoculated into ducklings through the leg. Here, the highest dose that did not affect the health of ducklings was chosen for immunization. Unexpectedly, at days 7 and 14 after immunization, the IgY titers of these ducklings were higher than those of ducklings immunized with the inactivated vaccine. However, the inactivated vaccine elicited higher IgY titers than the mutant strain after day 21. These findings suggest that the live bacteria stimulated the immune system of ducks more quickly than the inactivated vaccine, although the rate of protection was lower than that when the ducks were immunized with the inactivated vaccine. As a next step, the RA-CH-1*ΔB739_1343* mutant could be combined with adjuvant or immunopotentiator to increase the potential protection. In addition, it will be interesting to challenge with heterologous serotypes to determine whether the candidate vaccine protects against one or multiple different serotypes. Studies should also evaluate whether the RA-CH-1*ΔB739_1343* mutant stimulates the cell-mediated immune response to obtain further insight into the mechanism of protection. This work is the first to report a live attenuated *R*. *anatipestifer* vaccine candidate harboring a deletion in a gene involved in iron homeostasis.

In summary, compared with the wild-type strain RA-CH-1, *R*. *anatipestifer* CH-1 lacking *B739_1343* is significantly deficient in growth under iron-starved conditions. The *B739_1343* gene is required for bacterial virulence, and the RA-CH-1*ΔB739_1343* mutant can be used as a live attenuated vaccine for protecting ducks against *R*. *anatipestifer* CH-1.

## Materials and methods

### Bacterial strains, plasmids and primers

The bacterial strains and plasmids used in this study are described in Table 3. The primers used in this study are described in S1 Table.

**Table 3:** Strains and plasmids used in this study.

Strains and plasmids	Genotype or serotype	Source or reference
**Strains**		
XL1-BLUE	F- supE44 hdsR17 recA1 endA1 gyrA46 thi relA1 lac- F’ proAB- lacIq lacZΔM15 Tn10, Tet^r^	Laboratory collection
S17-1	Thi-1 thr leu tonA lac Y supE recA::RP4-2-Tc::Mu Kan^r^	[29]
S17-1 pEX18GM::*B739_1343*usd	S17-1 carrying pEX18GM::*B739_1343usd*, Kan^r^, Gen^r^	This study
RA-CH-1	*R*. *anatipestifer* serotype 1	Laboratory collection
RA-CH-1*ΔB739_1343*	*R*. *anatipestifer* CH-1 B739_1343::*spcR*, Spc^r^	This study
RA-CH-1*ΔB739_1343* pLMF03::*B739_1343*	*R*. *anatipestifer* CH-1 *B739_1343*::*spcR* carrying pLMF03::*B739_1343*	This study
**Plasmids**		
pEX18GM	oriT+, sacB+, gene replacement vector with MCS from pUC18, Gen^r^	[30]
pAM238	pSC101 origin, Spc^r^	[31]
pEX18GM::*B739_1343*usd	pEX18GM carrying *B739_1343usd* from *R*. *anatipestifer* CH-1 Gen^r^	This study
pLMF03	*B739_0921* promoter, oriColE1, ori pRA0726, Amp^r^, Cfx^r^	[32]
pLMF03::*B739_1343*	pLMF03 carrying *B739_1343* from *R*. *anatipestifer* CH-1, Amp^r^, Cfx^r^	This study

Amp^r^, ampicillin resistance; Gen^r^, gentamicin resistance; Kan^r^, kanamycin resistance; Spc^r^, spectinomycin resistance; Cfx^r^, cefoxitin resistance.

### Media and growth conditions

The preparation of 2,2’-dipyridyl (Dip) (Sigma–Aldrich, St. Louis, MO, USA) has been described elsewhere [32]. *E*. *coli* strains were grown on LB medium (Sigma-Aldrich, Product Number: L3522) at 37°C. Solid media contained 1.5% agar (Difco). The *R*. *anatipestifer* strains were grown on LB plates supplemented with 5% defibrinated sheep blood or TSA plates (tryptone soy broth, TSB, containing 1.5% agar) at 37°C. Iron-depleted medium was obtained by the addition of Dip. Antibiotics were added at the following final concentrations: ampicillin (Amp): 100 μg/mL, kanamycin (Kan): 50 μg/mL, and gentamicin (Gen): 20 μg/mL, for *E*. *coli*; and spectinomycin (Spec): 80 μg/mL, and cefoxitin (CfxA): 1 μg/mL for *R*. *anatipestifer* CH-1.

### Construction of the *B739_1343* gene deletion in *R*. *anatipestifer* CH-1

The *B739_1343* gene of *R*. *anatipestifer* CH-1 was deleted via allelic exchange through a recombinant suicide vector, pEX18GM [30]. Here, the *B739_1343* gene was replaced with a 1140-bp SpcR cassette according to a method described elsewhere [33]. Briefly, the 796-bp left flanking sequence and the 803-bp right flanking sequence of the *B739_1343* gene of *R*. *anatipestifer* CH-1 were amplified via PCR using the primer pairs B739_1343upP1 plus B739_1343upP2 and B739_1343downP1 plus B739_1343downP2, respectively (S1 Table). The SpcR cassette was amplified from the plasmid pAM238 [31] using the primer pairs SpcRP1 plus SpcRP2. The three resultant PCR fragments (B739_1343 upstream, B739_1343 downstream and SpcR cassette) were ligated using overlap PCR and digested with KpnI and BamHI. The resultant fragments were cloned into the pEX18GM plasmid to generate pEX18GM::*B739_1343usd*. Then, pEX18GM::*B739_1343usd* was further introduced into cells of the CaCl_2_-competent *E*. *coli* strain S17-1. pEX18GM::*B739_1343usd* was transferred to the recipient strain *R*. *anatipestifer* CH-1 through conjugation as described elsewhere [33]. The transconjugants were screened using blood agar plates supplemented with Kan (50 μg/mL) and Spec (80 μg/mL). The gene deletion mutant strains were identified by PCR by amplifying the conserved 16S rRNA gene of *R*. *anatipestifer* using the primers 16S rRNA P1 and 16S rRNA P2 and the deleted gene using the corresponding primers B739_1343compP1 and B739_1343compP2 (S3 Fig).

### Construction of the RA-CH-1*ΔB739_1343* pLMF03::*B739_1343* complementation strain

To construct the RA-CH-1*ΔB739_1343* complementation strain, the *B739_1343* gene of *R*. *anatipestifer* CH-1, with its own promoter, was amplified using the primers B739_1343compP1 and B739_1343compP2, which contained SalI and XbaI restriction sites, respectively (S1 Table). The fragments were cut by these two enzymes and cloned into the shuttle plasmid pLMF03 [32]. The resulting plasmid, pLMF03::*B739_1343*, was transformed into cells of the CaCl_2_-competent strain *E*. *coli* S17-1, and the recombinant plasmid was introduced into the RA-CH-1*ΔB739_1343* mutant strain via conjugation as described elsewhere [32]. The transconjugants were selected using blood agar plates supplemented with Cfx (1 μg/mL) and Kan (50 μg/mL) and identified by PCR amplification of 16S rRNA and *B739_1343* using the primer pairs 16S rRNA P1 plus 16S rRNA P2 and B739_1343compP1 plus B739_1343compP2, respectively (S1 Table and S3 Fig). The resulting strain was designated RA-CH-1*ΔB739_1343*pLMF03::*B739_1343*.

### *In vitro* growth rate determination

The *in vitro* growth rates of the test strains were determined by measuring the optical density (OD) at 600 nm with a spectrophotometer (Eppendorf Biophotometer, Germany). Briefly, early exponential-phase cultures were inoculated into 20 mL of TSB or TSB supplemented with Dip (120 μM), Dip (120 μM) and Fe(NO_3_)_3_ (300 μM) at OD600 0.1, followed by incubation at 37°C with shaking at 180 rpm. The OD was determined at 600 nm every 2 h for 14 h. The experiment was performed using three independent experiments with two replicate samples for each experiment.

### Iron utilization experiment on TSA plates

RA-CH-1pLMF03, RA-CH-1*ΔB739_1343*pLMF03 and RA-CH-1*ΔB739_1343* pLMF03::*B739_1343* were inoculated onto 5% sheep blood plates, which were then incubated overnight at 37°C. The bacterial strains were subsequently collected, re-suspended in 1 mL of PBS and centrifuged for 5 min at 6,000 rpm. This operation was repeated three times to wash the bacteria. The OD_600_ values of the bacterial suspensions were then checked and adjusted to OD_600_ = 1. Next, the standardized strains were diluted to 10^4^ bacteria/mL (1OD_600_ = 6×10^8^ bacteria), and a 20-μL (approximately 200 bacteria) sample of each strain was inoculated onto a TSA plate or a TSA plate containing 50 μM Dip. Growth was recorded after a 2-day incubation at 37°C.

### qRT-PCR

RA-CH-1 was inoculated into 20 mL of TSB medium and 20 mL of TSB medium with 200 μM Dip at an OD600 of 0.05 at 37°C (the glassware was deferrated by acid-washing). After 6–8 h of incubation (corresponding to mid-log growth phase), the bacteria were immediately mixed with a two-fold volume of RNA protect Bacteria Reagent (Qiagen: 76506) and centrifuged again at 5,000 g for 10 min. RNA extraction and reverse transcription were performed as described elsewhere [32]. qPCR was conducted using SYBR Green Master Mix (Vazyme:Q111-01) and primers at 0.2 μM. Each experiment consisted of three biological replicate samples with three technical replicates each. The fold change was calculated as described in reference [34] with the delta delta Ct method to consider the efficiency of the PCR reaction for each target, and *recA* served as the reference gene [32].

### LD_50_ determination

The bacterial LD_50_ was measured to evaluate virulence as previously described [35]. Briefly, for each strain, 3-day-old Pekin ducks were randomly divided into four groups (10 ducks/group). The ducks were then injected intramuscularly with 10^7^, 10^8^, 10^9^, or 10^10^ CFU of each bacterial strain and were examined every 4–6 hours for seven days. Once the ducks exhibited signs of moribundity, including depression, lack of movement or refusal of food, they were euthanized via forced inhalation of CO_2_, and identification of *R*. *anatipestifer* was subsequently performed. The mortality of the ducks was recorded daily for seven days post-challenge. The LD_50_ was calculated using the Reed-Muench method [36].

### *In vivo* competition assay

A competition assay was performed *in vivo* as previously described by Hagan [16] and Wang et al [15]. Briefly, bacterial strains were grown to exponential phase in TSB medium and collected via centrifugation at 6,000 g. This operation was repeated three times to wash the bacteria. The re-suspended strains were adjusted to 10^10^ CFU per mL. The standardized RA-CH-1 and RA-CH-1*ΔB739_1343* mutant strains were mixed at a 1:1 ratio, and 200 μL of the mixture containing 10^9^ CFU of each strain was injected intramuscularly into the same 3-day-old Pekin ducks. At 24 h and 48 h post-inoculation, the ducks were euthanized by forced inhalation of CO_2_. Heart blood, liver and brain tissue were collected at 24 h and 48 h post-inoculation (from six ducks at each time point). Liver and brain samples were weighed and homogenized in PBS. Dilutions of heart blood and the homogenates were then plated on TSA agar to determine the CFU of bacteria per mL of heart blood or per gram of tissue. Additionally, dilutions of heart blood and homogenate were also plated on TSA agar containing Spec (80 μg/mL) to differentiate RA-CH-1 and RA-CH-1*ΔB739_1343*.

### Immunization and challenge

To investigate whether RA-CH-1*ΔB739_1343* could be used as an attenuated live vaccine candidate, the RA-CH-1*ΔB739_1343* strain and an inactivated RA-CH-1 vaccine (Chengdu Tecbond Biological Products Corporation, Sichuan, China) were prepared for immunization. Briefly, the mutant strain RA-CH-1*ΔB739_1343* was cultured in TSB at 37°C to exponential phase. Then, RA-CH-1*ΔB739_1343* was collected and re-suspended in PBS. The re-suspended strain was adjusted to 5×10^8^ CFU per mL. Each duck was intramuscularly injected with 200 μL of the standardized strain RA-CH-1*ΔB739_1343* containing 10^8^ CFU of bacterial cells. The inactivated RA-CH-1 vaccine was subcutaneously injected, as a control, into the neck according to the manufacturer’s recommendations.

Three-day-old Pekin ducks were randomly divided into four groups (twenty ducks per group). Group 1 was injected with PBS; group 2 was immunized with RA-CH-1*ΔB739_1343*; and group 3 was immunized with the inactivated RA-CH-1 vaccine. Group 4 was not subjected to immunization and challenge and was used as a control. To evaluate the safety of RA-CH-1*ΔB739_1343*, the habits, appetites, mental status and other clinical manifestations of the vaccinated ducks were observed for 12 days post-vaccination. Furthermore, the average bodyweights of the ducklings in all four groups were recorded every three days until challenge.

On day 12 after immunization, the ducks in groups 1, 2 and 3 were challenged with wild-type *R*. *anatipestifer* CH-1 via intramuscular injection at a dose of 2.28×10^10^ CFU (100-fold LD_50_) per duck. Deaths were recorded, and clinical manifestations were observed daily for 10 days after challenge to evaluate the protection rate of the vaccine. Similarly, moribund ducks were euthanized by forced inhalation of CO_2_. The protection rate was calculated as described by Sandhu [37] with some modification as follows: [1−(%Morbidity in vaccinated/%Morbidity in control)]×100.

### Determination of serum antibody titers via ELISA

A total of 10 3-day-old Pekin ducks were randomly assigned to two groups (five ducks per group) and immunized intramuscularly with RA-CH-1*ΔB739_1343* or subcutaneously in the neck with the inactivated RA-CH-1 vaccine. Blood samples were collected before vaccination (D0) as a control and weekly thereafter until D49 after primary immunization.

For antigen preparation, cultured *R*. *anatipestifer* CH-1 was grown in TSB medium to exponential phase, then harvested via centrifugation at 8,000 g for 10 min at 4°C and washed twice with PBS buffer. The bacterial pellets were re-suspended in 25 mL of buffer (20 mM Tris–HCl pH 7.4, 10 mM EDTA, 1 mM TLCK) and lysed using a French press. Cellular debris was removed via centrifugation at 8,000×g for 30 min at 4°C, and the protein concentration of the *R*. *anatipestifer* CH-1 lysate was determined using a BCA Protein Assay Kit (Thermo Scientific, USA). ELISA was performed as described previously using 1 μg/well of *R*. *anatipestifer* CH-1 [23]. Briefly, 96-well ELISA plates were coated with the *R*. *anatipestifer* CH-1 lysate antigen in 100 μL of bicarbonate buffer (pH 9.6) and incubated at 4°C overnight. The plates were then washed with PBS containing 0.1% Tween-20 (PBST) three times and blocked with 1% BSA in PBST at 37°C for 1 h. After blocking, serial-diluted duck serum (from 1:20 to 1:20,480) was added to the wells, followed by incubation at 37°C for 2 h. Thereafter, the plates were washed three times with PBST, and horseradish peroxidase (HRP)-conjugated goat anti-duck IgY (1:5,000 dilution) (ab112771, Abcam) was added. The plates were then incubated at 37°C for 1 h, washed three times with PBST, and 100 μL of soluble TMB substrate solution (TIANGEN, China) was added to each well. The reaction was stopped by adding 100 μL of 2 M H_2_SO_4_, and the plates were read at 450 nm using a 680 microplate reader (Bio-Rad, USA). The highest dilutions of the sera with an OD_450_ value 2.1 times that of the negative control wells were used as the ELISA titers. The experiment was performed using three independent experiments with three replicates samples for each experiment.

### Animals and ethics statement

One-day-old Pekin ducks were purchased from Grimaud farms in Chengdu (Sichuan, China) and housed at our animal facilities with free access to food and water.

This study was performed in accordance with the recommendations of the local animal welfare bodies and the Sichuan Agricultural University ethics committee (SYXK2014-187). The protocol was approved by the Sichuan Agricultural University ethics committee.

### Sequence analysis

The homology of the B739_1343 sequences was analyzed using the Basic Local Alignment Search Tool (BLAST) algorithm (http://blast.ncbi.nlm.nih.gov/). Prediction of protein structure was performed using Phyre^2^ programme online (http://www.sbg.bio.ic.ac.uk/phyre2/html/page.cgi?id=index) [38]. Multiple sequence alignments of the B739_1343 sequences were performed using the program Clustal W2 [39].

### Statistical analysis

Statistical analysis was performed using GraphPad Prism 6 software and SPSS statistics 20 for Windows. The statistical significance of the data was ascertained using Student’s T test. A value of P<0.05 was considered significant.

## Supporting information

S1 File ARRIVE checklist. (PDF)

S1 Fig The transcriptional levels of *B739_1342* in RA-CH-1*ΔB739_1343* and RA-CH-1. Quantitative real-time PCR analysis of the relative expression of *B739_1342* in RA-CH-1*ΔB739_1343* and RA-CH-1 in TSB. The fold change was calculated with the delta delta Ct method to consider the efficiency of the PCR reaction for each target gene. The error bars represent the standard deviations of three independent experiments (n = 3).
(TIF)

S2 Fig The fold change in the transcriptional levels of RA-CH-1 *B739_1343* and *B739_0103* in TSB and TSB supplemented with 200 μM Dip. Quantitative real-time PCR analysis of the relative expression of RA-CH-1 *B739_1343* (A) and *B739_0103* (B) mRNA in TSB and in TSB supplemented with 200 μM Dip. The fold change was calculated with the delta delta Ct method to consider the efficiency of the PCR reaction for each target. The error bars represent the standard deviations of three independent experiments (n = 3).
(TIF)

S3 Fig Characterization of the *R*. *anatipestifer* CH-1 mutant strain (RA-CH-1*ΔB739_1343*) and the complementation strain (RA-CH-1*ΔB739_1343*pLMF03::*B739_1343*) by PCR. (A) Verification of the deletion of *B739_1343* by PCR. Lane M, BM5000 DNA Marker (Biomed, Beijing, China). Lane 1 and Lane 2: 16S rRNA (960 bp) was amplified from RA-CH-1 and RA-CH-1*ΔB739_1343* using the primers 16S rRNA P1 and 16S rRNA P2, respectively. Lanes 3–5: The SpcR cassette (1140 bp) was amplified from the plasmid pAM238, RA-CH-1 and RA-CH-1*ΔB739_1343* using the primers SpcR P1 and SpcR P1, respectively. Lane 6 and Lane 7: The *B739_1343* gene (2352 bp) was amplified from RA-CH-1 and RA-CH-1*ΔB739_1343* using the primers B739_1343compP1 and B739_1343compP2, respectively. Lane 8, Lane 9 and Lane 10: The *sacB* gene (1422 bp) was amplified from the plasmid pEX18GM, RA-CH-1 and RA-CH-1*ΔB739_1343* using the primers SacB P1 and SacB P2, respectively. (B) Verification of the complementation strain RA-CH-1*ΔB739_1343*pLMF03::*B739_1343* by PCR. Lane M: BM5000 DNA Marker (Biomed, Beijing, China). Lane 1: 16S rRNA (960 bp). Lane 2: *B739_1343* gene (2352 bp). Lane 3: SpcR cassette (1140 bp). Lane 4: CfxA resistance gene (638 bp).
(TIF)

S1 Table Primers used in this study. (DOCX)

## References

[1] Hancock RE, Hantke K, Braun V. Iron transport of *Escherichia coli* K-12: involvement of the colicin B receptor and of a citrate-inducible protein. Journal of bacteriology. 1976;127(3):1370–5. . 783142 10.1128/jb.127.3.1370-1375.1976 PMC232932

[2] Hancock RE, Hantke K, Braun V. Iron transport in *Escherichia coli* K-12. 2,3-Dihydroxybenzoate-promoted iron uptake. Archives of microbiology. 1977;114(3):231–9. . 143918 10.1007/BF00446867

[3] Cherayil BJ. The role of iron in the immune response to bacterial infection. Immunologic research. 2011;50(1):1–9. doi: 10.1007/s12026-010-8199-1 21161695 10.1007/s12026-010-8199-1 PMC3085559

[4] Cassat JE, Skaar EP. Iron in infection and immunity. Cell host & microbe. 2013;13(5):509–19. doi: 10.1016/j.chom.2013.04.010 23684303 10.1016/j.chom.2013.04.010 PMC3676888

[5] Miethke M, Marahiel MA. Siderophore-based iron acquisition and pathogen control. Microbiology and molecular biology reviews: MMBR. 2007;71(3):413–51. doi: 10.1128/MMBR.00012-07 . 17804665 10.1128/MMBR.00012-07 PMC2168645

[6] Contreras H, Chim N, Credali A, Goulding CW. Heme uptake in bacterial pathogens. Current opinion in chemical biology. 2014;19:34–41. doi: 10.1016/j.cbpa.2013.12.014 . 24780277 10.1016/j.cbpa.2013.12.014 PMC4007353

[7] Segers P, Mannheim W, Vancanneyt M, De Brandt K, Hinz KH, Kersters K, et al *Riemerella anatipestifer* gen. nov., comb. nov., the causative agent of septicemia anserum exsudativa, and its phylogenetic affiliation within the Flavobacterium-Cytophaga rRNA homology group. Int J Syst Bacteriol. 1993;43 doi: 10.1099/00207713-43-4-768 8240957 10.1099/00207713-43-4-768

[8] Ruiz JA, Sandhu TS. *Riemerella anatipestifer* infection In: Swayne DE, Glisson JR, McDougald LR, Nolan LK, Suarez DL, Nair VL, editors. Diseases of Poultry. 13th Edition ed: John Wiley & Sons, Inc., Hoboken, New Jersey, USA; 2013 p. 823–8.

[9] Sandhu TS, Leister ML. Serotypes of *'Pasteurella'* anatipestifer isolates from poultry in different countries. Avian pathology: journal of the WVPA. 1991;20(2):233–9. doi: 10.1080/03079459108418760 . 18680018 10.1080/03079459108418760

[10] Loh H, Teo TP, Tan HC. Serotypes of *'Pasteurella'* anatipestifer isolates from ducks in Singapore: a proposal of new serotypes. Avian pathology: journal of the WVPA. 1992;21(3):453–9. doi: 10.1080/03079459208418863 . 18670960 10.1080/03079459208418863

[11] Pathanasophon P, Sawada T, Tanticharoenyos T. New serotypes of *Riemerella anatipestifer* isolated from ducks in Thailand. Avian pathology: journal of the WVPA. 1995;24(1):195–9. doi: 10.1080/03079459508419059 . 18645776 10.1080/03079459508419059

[12] Guo Y, Hu D, Guo J, Li X, Guo J, Wang X, et al The Role of the Regulator Fur in Gene Regulation and Virulence of *Riemerella anatipestifer* Assessed Using an Unmarked Gene Deletion System. Frontiers in cellular and infection microbiology. 2017;7:382 doi: 10.3389/fcimb.2017.00382 . 28971067 10.3389/fcimb.2017.00382 PMC5609570

[13] Zheng F, Lin G, Zhou J, Wang G, Cao X, Gong X, et al Loop-mediated isothermal amplification assay targeting the *ompA* gene for rapid detection of *Riemerella anatipestifer*. Mol Cell Probes. 2011;25(1):65–7. doi: 10.1016/j.mcp.2010.10.004 . 21040782 10.1016/j.mcp.2010.10.004

[14] Lu F, Miao S, Tu J, Ni X, Xing L, Yu H, et al The role of TonB-dependent receptor TbdR1 in *Riemerella anatipestifer* in iron acquisition and virulence. Veterinary microbiology. 2013;167(3–4):713–8. doi: 10.1016/j.vetmic.2013.08.020 . 24075356 10.1016/j.vetmic.2013.08.020

[15] Wang M, Zhang P, Zhu D, Wang M, Jia R, Chen S, et al Identification of the ferric iron utilization gene *B739_1208* and its role in the virulence of *R*. *anatipestifer* CH-1. Veterinary microbiology. 2017;201:162–9. doi: 10.1016/j.vetmic.2017.01.027 . 28284604 10.1016/j.vetmic.2017.01.027

[16] Hagan EC, Mobley HL. Haem acquisition is facilitated by a novel receptor Hma and required by uropathogenic *Escherichia coli* for kidney infection. Molecular microbiology. 2009;71(1):79–91. doi: 10.1111/j.1365-2958.2008.06509.x . 19019144 10.1111/j.1365-2958.2008.06509.x PMC2736550

[17] Braun V, Hantke K. Recent insights into iron import by bacteria. Current opinion in chemical biology. 2011;15(2):328–34. doi: 10.1016/j.cbpa.2011.01.005 . 21277822 10.1016/j.cbpa.2011.01.005

[18] Datta S, Crosa JH. Identification and characterization of a novel outer membrane protein receptor required for hemin utilization in *Vibrio vulnificus*. Biometals: an international journal on the role of metal ions in biology, biochemistry, and medicine. 2012;25(2):275–83. doi: 10.1007/s10534-011-9501-y . 22015545 10.1007/s10534-011-9501-y PMC3288223

[19] Allen CE, Schmitt MP. HtaA is an iron-regulated hemin binding protein involved in the utilization of heme iron in *Corynebacterium diphtheriae*. Journal of bacteriology. 2009;191(8):2638–48. doi: 10.1128/JB.01784-08 . 19201805 10.1128/JB.01784-08 PMC2668399

[20] Porcheron G, Dozois CM. Interplay between iron homeostasis and virulence: Fur and RyhB as major regulators of bacterial pathogenicity. Veterinary microbiology. 2015;179(1–2):2–14. doi: 10.1016/j.vetmic.2015.03.024 . 25888312 10.1016/j.vetmic.2015.03.024

[21] Cornelis P, Wei Q, Andrews SC, Vinckx T. Iron homeostasis and management of oxidative stress response in bacteria. Metallomics: integrated biometal science. 2011;3(6):540–9. doi: 10.1039/c1mt00022e . 21566833 10.1039/c1mt00022e

[22] Liu M, Huang M, Zhu D, Wang M, Jia R, Chen S, et al Identifying the Genes Responsible for Iron-Limited Condition in *Riemerella anatipestifer* CH-1 through RNA-Seq-Based Analysis. BioMed research international. 2017;2017:8682057 doi: 10.1155/2017/8682057 . 28540303 10.1155/2017/8682057 PMC5429918

[23] Lee JW, Lin YM, Yen TY, Yang WJ, Chu CY. CpG oligodeoxynucleotides containing GACGTT motifs enhance the immune responses elicited by a goose parvovirus vaccine in ducks. Vaccine. 2010;28(50):7956–62. doi: 10.1016/j.vaccine.2010.09.072 . 20933041 10.1016/j.vaccine.2010.09.072

[24] Fusco WG, Choudhary NR, Council SE, Collins EJ, Leduc I. Mutational Analysis of Hemoglobin Binding and Heme Utilization by a Bacterial Hemoglobin Receptor. Journal of bacteriology. 2013;195(13):3115–23. doi: 10.1128/JB.00199-13 23667232 10.1128/JB.00199-13 PMC3697543

[25] Payne SM, Wyckoff EE, Murphy ER, Oglesby AG, Boulette ML, Davies NM. Iron and pathogenesis of *Shigella*: iron acquisition in the intracellular environment. Biometals: an international journal on the role of metal ions in biology, biochemistry, and medicine. 2006;19(2):173–80. doi: 10.1007/s10534-005-4577-x . 16718602 10.1007/s10534-005-4577-x

[26] Fetherston JD, Mier I Jr., Truszczynska H, Perry RD. The Yfe and Feo transporters are involved in microaerobic growth and virulence of *Yersinia pestis* in bubonic plague. Infection and immunity. 2012;80(11):3880–91. doi: 10.1128/IAI.00086-12 . 22927049 10.1128/IAI.00086-12 PMC3486059

[27] Watson RJ, Millichap P, Joyce SA, Reynolds S, Clarke DJ. The role of iron uptake in pathogenicity and symbiosis in *Photorhabdus luminescens* TT01. BMC microbiology. 2010;10:177 doi: 10.1186/1471-2180-10-177 . 20569430 10.1186/1471-2180-10-177 PMC2905363

[28] Tu J, Lu F, Miao S, Ni X, Jiang P, Yu H, et al The siderophore-interacting protein is involved in iron acquisition and virulence of *Riemerella anatipestifer* strain CH3. Veterinary microbiology. 2014;168(2–4):395–402. doi: 10.1016/j.vetmic.2013.11.027 . 24345412 10.1016/j.vetmic.2013.11.027

[29] Simon R, Priefer U, P A, uuml, hler. A Broad Host Range Mobilization System for In Vivo Genetic Engineering: Transposon Mutagenesis in Gram Negative Bacteria. Nature Biotechnology. 1983;1(9):784–91.

[30] Hoang TT, Karkhoff-Schweizer RR, Kutchma AJ, Schweizer HP. A broad-host-range Flp-FRT recombination system for site-specific excision of chromosomally-located DNA sequences: application for isolation of unmarked *Pseudomonas aeruginosa* mutants. Gene. 1998;212(1):77–86. . 9661666 10.1016/s0378-1119(98)00130-9

[31] Rossi MS, Paquelin A, Ghigo JM, Wandersman C. Haemophore-mediated signal transduction across the bacterial cell envelope in *Serratia marcescens*: the inducer and the transported substrate are different molecules. Molecular microbiology. 2003;48(6):1467–80. . 12791131 10.1046/j.1365-2958.2003.03516.x

[32] Liu M, Wang M, Zhu D, Wang M, Jia R, Chen S, et al Investigation of TbfA in *Riemerella anatipestifer* using plasmid-based methods for gene over-expression and knockdown. Scientific reports. 2016;6:37159 doi: 10.1038/srep37159 . 27845444 10.1038/srep37159 PMC5109031

[33] Liao H, Cheng X, Zhu D, Wang M, Jia R, Chen S, et al TonB Energy Transduction Systems of *Riemerella anatipestifer* Are Required for Iron and Hemin Utilization. PloS one. 2015;10(5):e0127506 doi: 10.1371/journal.pone.0127506 . 26017672 10.1371/journal.pone.0127506 PMC4446302

[34] Pfaffl MW. A new mathematical model for relative quantification in real-time RT-PCR. Nucleic acids research. 2001;29(9):e45 . 11328886 10.1093/nar/29.9.e45 PMC55695

[35] Zou J, Wang X, Tian M, Cao S, Hou W, Wang S, et al The M949_1556 gene plays a role on the bacterial antigenicity and pathogenicity of *Riemerella anatipestifer*. Veterinary microbiology. 2015;177(1–2):193–200. doi: 10.1016/j.vetmic.2015.03.003 . 25804836 10.1016/j.vetmic.2015.03.003

[36] Reed L, Muench H. A simple method of estimating fifty per cent endpoints. Am J Hyg. 1938;27(3):493–7.

[37] Sandhu T. Immunization of White Pekin Ducklings against *Pasteurella anatipestifer* Infection. Avian Diseases. 1979;23(3):662–9. doi: 10.2307/1589742 526203

[38] Kelley LA, Mezulis S, Yates CM, Wass MN, Sternberg MJ. The Phyre2 web portal for protein modeling, prediction and analysis. Nat Protoc. 2015;10(6):845–58. doi: 10.1038/nprot.2015.053 . 25950237 10.1038/nprot.2015.053 PMC5298202

[39] Larkin MA, Blackshields G, Brown NP, Chenna R, McGettigan PA, McWilliam H, et al Clustal W and Clustal X version 2.0. Bioinformatics. 2007;23(21):2947–8. doi: 10.1093/bioinformatics/btm404 . 17846036 10.1093/bioinformatics/btm404

