# Supplementary figures and images for: Roles of B739_1343 in iron acquisition and pathogenesis in Riemerella anatipestifer CH-1 and evaluation of the RA-CH-1ΔB739_1343 mutant as an attenuated vaccine
Source: PLoS One. 2018 May 30;13(5):e0197310. doi: 10.1371/journal.pone.0197310 (PMC5976166; doi:10.1371/journal.pone.0197310)

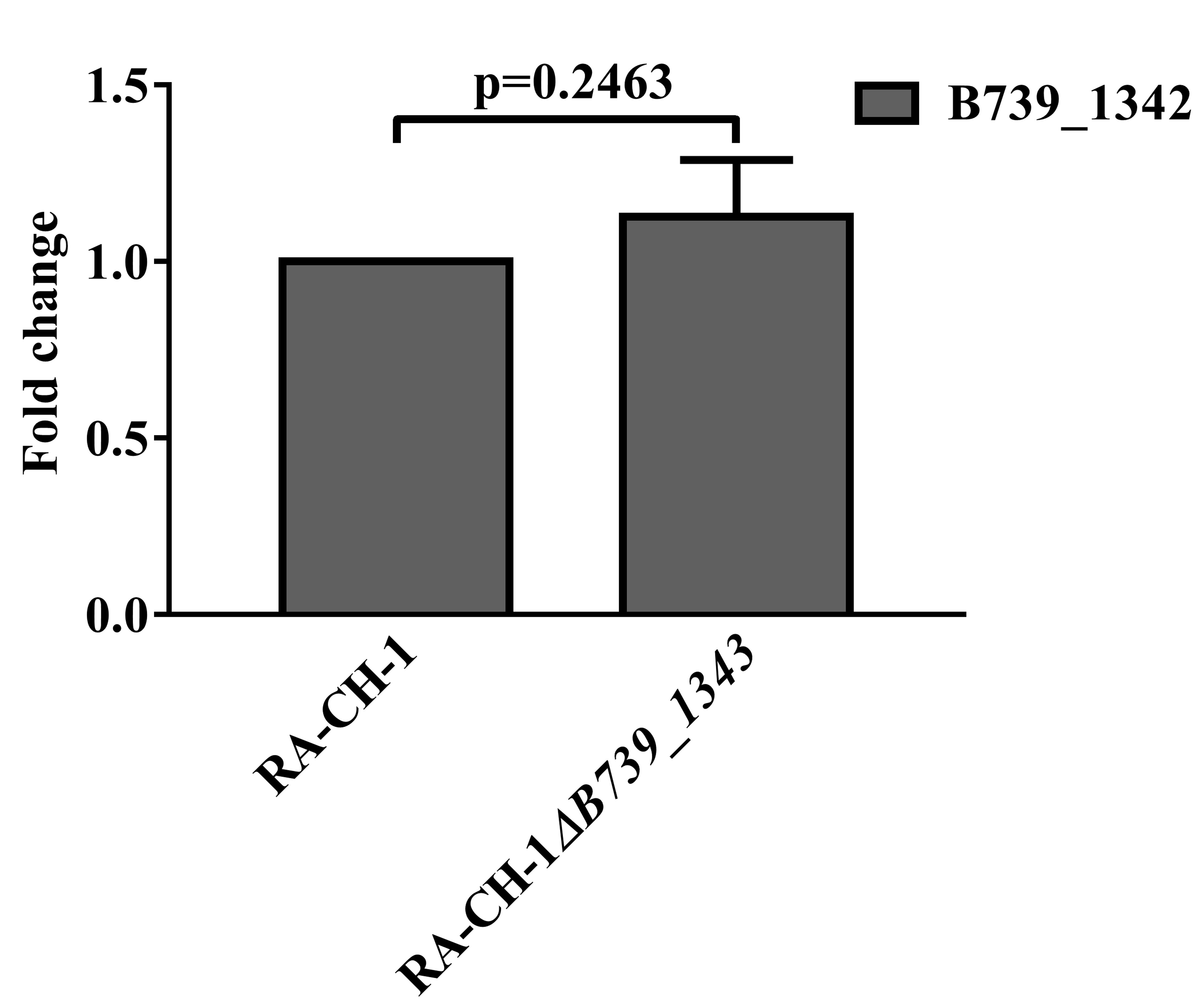

Supplement: S1 Fig — Quantitative real-time PCR analysis of the relative expression of B739_1342 in RA-CH-1ΔB739_1343 and RA-CH-1 in TSB. The fold change was calculated with the delta delta Ct method to consider the efficiency of the PCR reaction for each target gene. The error bars represent the standard deviations of three independent experiments (n = 3). (TIF) [file pone.0197310.s002.tif]

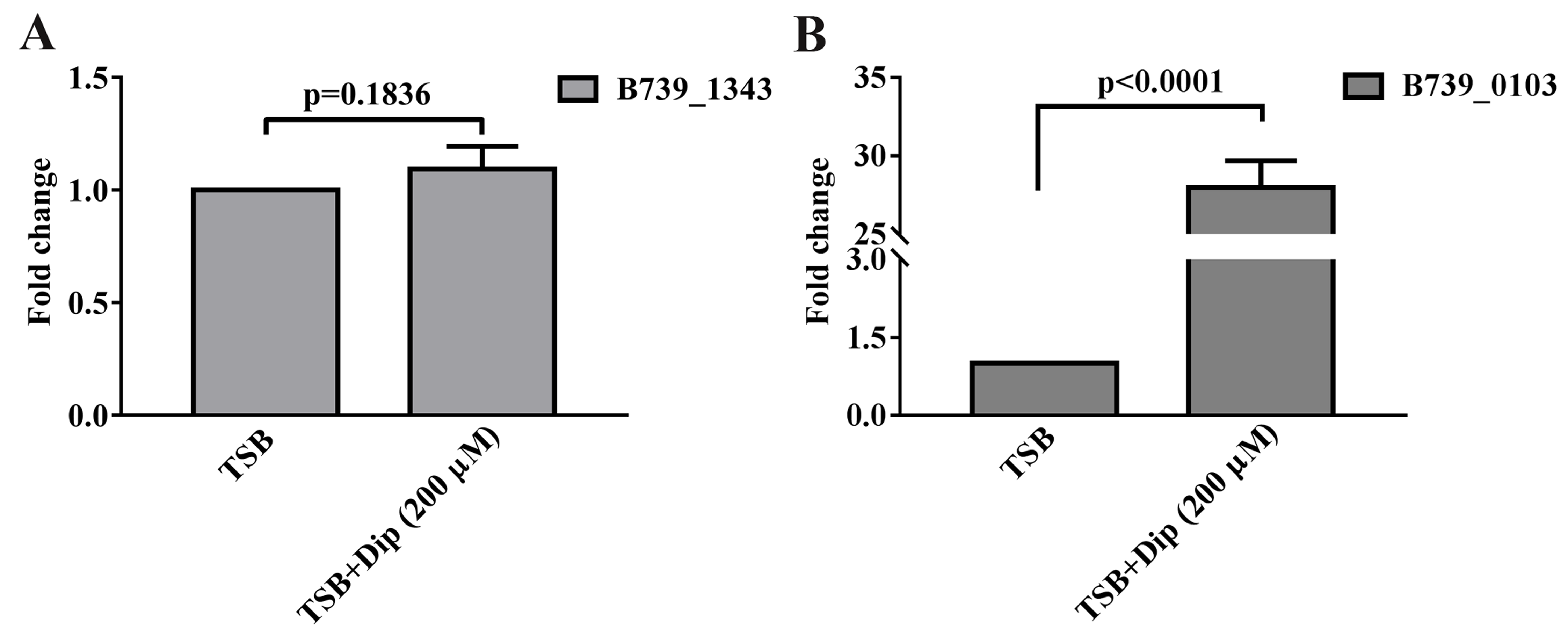

Supplement: S2 Fig — Quantitative real-time PCR analysis of the relative expression of RA-CH-1 B739_1343 (A) and B739_0103 (B) mRNA in TSB and in TSB supplemented with 200 μM Dip. The fold change was calculated with the delta delta Ct method to consider the efficiency of the PCR reaction for each target. The error bars represent the standard deviations of three independent experiments (n = 3). (TIF) [file pone.0197310.s003.tif]

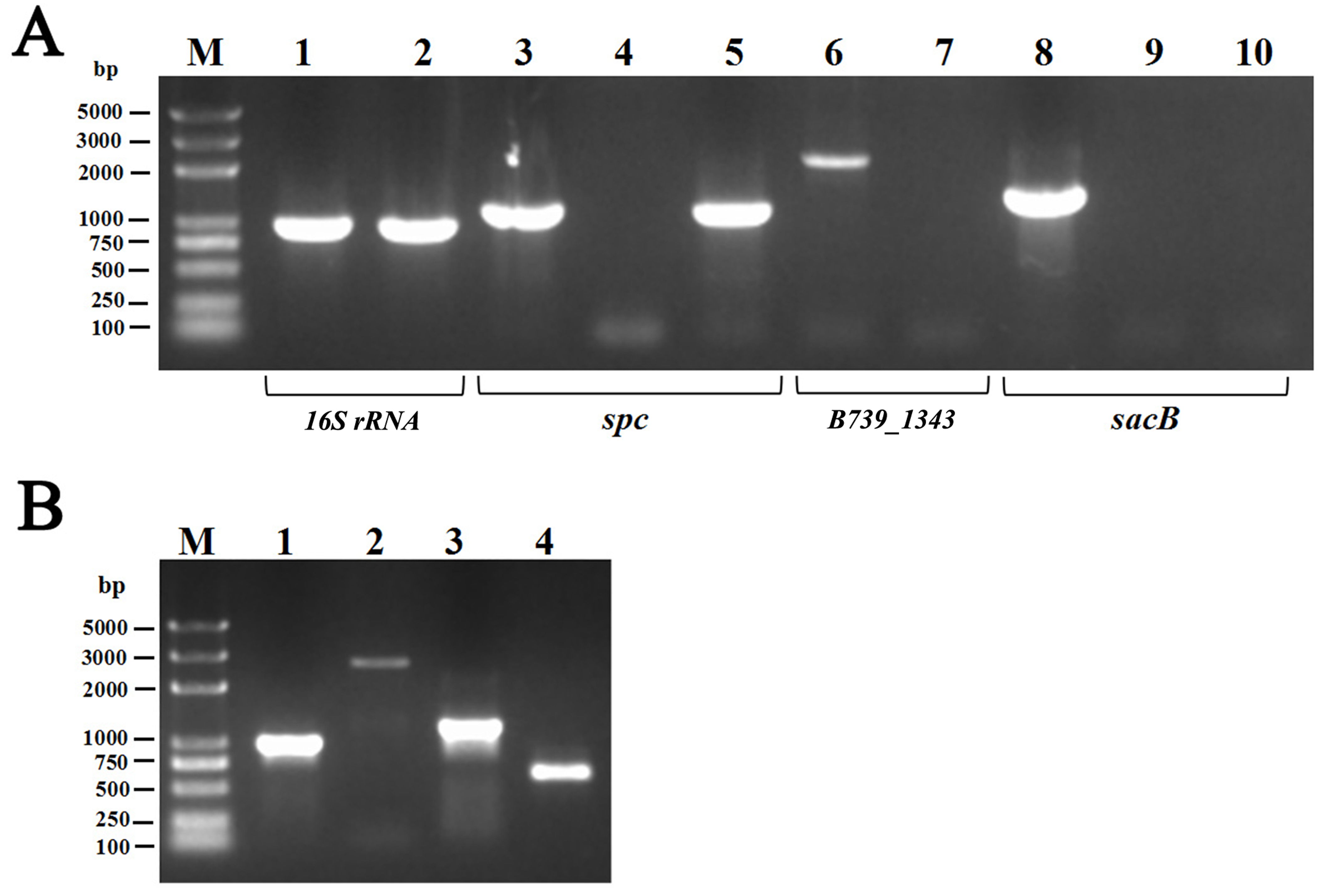

Supplement: S3 Fig — (A) Verification of the deletion of B739_1343 by PCR. Lane M, BM5000 DNA Marker (Biomed, Beijing, China). Lane 1 and Lane 2: 16S rRNA (960 bp) was amplified from RA-CH-1 and RA-CH-1ΔB739_1343 using the primers 16S rRNA P1 and 16S rRNA P2, respectively. Lanes 3–5: The SpcR cassette (1140 bp) was amplified from the plasmid pAM238, RA-CH-1 and RA-CH-1ΔB739_1343 using the primers SpcR P1 and SpcR P1, respectively. Lane 6 and Lane 7: The B739_1343 gene (2352 bp) was amplified from RA-CH-1 and RA-CH-1ΔB739_1343 using the primers B739_1343compP1 and B739_1343compP2, respectively. Lane 8, Lane 9 and Lane 10: The sacB gene (1422 bp) was amplified from the plasmid pEX18GM, RA-CH-1 and RA-CH-1ΔB739_1343 using the primers SacB P1 and SacB P2, respectively. (B) Verification of the complementation strain RA-CH-1ΔB739_1343pLMF03::B739_1343 by PCR. Lane M: BM5000 DNA Marker (Biomed, Beijing, China). Lane 1: 16S rRNA (960 bp). Lane 2: B739_1343 gene (2352 bp). Lane 3: SpcR cassette (1140 bp). Lane 4: CfxA resistance gene (638 bp). (TIF) [file pone.0197310.s004.tif]
