# Supplementary material for: Roles of B739_1343 in iron acquisition and pathogenesis in Riemerella anatipestifer CH-1 and evaluation of the RA-CH-1ΔB739_1343 mutant as an attenuated vaccine
Source: PLoS One. 2018 May 30;13(5):e0197310. doi: 10.1371/journal.pone.0197310 (PMC5976166; doi:10.1371/journal.pone.0197310)
Supplement: S1 Table — (DOCX) [file pone.0197310.s005.docx]

**S1 Table. Primers used in this study**

| **Primer** | **Organism** | **Sequence** |
| --- | --- | --- |
| SpcRP1 | pAM238 | GAAATCAATCTGGATTGGACTCGACTTCGCTGCTGCCC |
| SpcRP2 | pAM238 | GTTTTAATTAAAAATGTTAATAGCGAATTGTTAGACATTATTTGC |
| B739_1343upP1 | *R. anatipestifer* CH-1 | CGGGATCCCGGTACCACCTTGGTTAATAATATTC |
| B739_1343upP2 | *R. anatipestifer* CH-1 | GGGCAGCAGCGAAGTCGAGTCCAATCCAGATTGATTTC |
| B739_1343downP1 | *R. anatipestifer* CH-1 | GCAAATAATGTCTAACAATTCGCTATTAACATTTTTAATTAAAAC |
| B739_1343downP2 | *R. anatipestifer* CH-1 | GGGGTACCCCTACTTAGAGAGCGTACAGCTCC |
| 16S rRNAP1 | *R. anatipestifer* CH-1 | CTTCGGATACTTGAGAGCG |
| 16S rRNAP2  SacBP1  SacBP2  B739_1343compP1  B739_1343compP2  CfxP1  CfxP2  B739_1343 qRTP1  B739_1343 qRTP2  B739_1342 qRTP1  B739_1342 qRTP2  B739_0103 qRTP1  B739_0103 qRTP2  RecA qRTP1  RecA qRTP2 | *R. anatipestifer* CH-1  pEX18GM  pEX18GM  *R. anatipestifer* CH-1  *R. anatipestifer* CH-1  pLMF03  pLMF03  *R. anatipestifer* CH-1  *R. anatipestifer* CH-1  *R. anatipestifer* CH-1  *R. anatipestifer* CH-1  *R. anatipestifer* CH-1  *R. anatipestifer* CH-1  *R. anatipestifer* CH-1  *R. anatipestifer* CH-1 | GCAGCACCTTGAAAATTGT  CATGCCATGGATGAACATCAAAAAGTTTGC  CTAGTCTAGACTAGTTATTTGTTAACTGTTAATTGTCCTTGTTCAAGG  ACGCGTCGACGTCGGCCATAGCGGCCGCGGAAGGCATTCTCTGCTTCCCG  CTAGTCTAGATTAAAAATTAAATTGACAAGTG  TTTCATTGTTCCATAAATCAGC  TACACCTGTTTTGCATTCTTTT  CTAAAGTACGGTGGAGATGC  TTTACCATTGCTGATGCCTG  CGACGAAGTACAATACCTTACC  TTTCATCAGTAACATCGTGGG  TAATACCAGCCGTACATTGC  TATTCAAGTTCCGTCTGCAC  TGAAACTAGGTGATGGTACG  CTTAGGATAACCGCCTACTC |
